# Antioxidant Activity of *Bougainvillea spectabilis* Bracts as an Alternative to Nitrites in Cooked Pork Ham

**DOI:** 10.3390/foods13193070

**Published:** 2024-09-26

**Authors:** T. Alexandra Ferreira, Jose A. Rodriguez, Irais Sánchez-Ortega, Jose M. Lorenzo, Eva M. Santos

**Affiliations:** 1 Campus Puebla, Universidad del Valle de Mexico, Camino Real a San Andrés Cholula No. 4002, Emiliano Zapata, San Andrés Cholula 72810, Mexico; thania.ferreira@uvmnet.edu; 2 Área Académica de Química, Universidad Autónoma del Estado de Hidalgo, Carr. Pachuca-Tulancingo Km. 4.5, Mineral de la Reforma 42184, Mexico; josear@uaeh.edu.mx (J.A.R.); irais_sanchez5498@uaeh.edu.mx (I.S.-O.); 3 Centro Tecnológico de la Carne de Galicia, Rúa Galicia n° 4, Parque Tecnológico de Galicia, San Cibrao das Viñas, 32900 Ourense, Spain; jmlorenzo@ceteca.net; 4 Área de Tecnoloxía dos Alimentos, Facultade de Ciencias, Universidade de Vigo, 32004 Ourense, Spain

**Keywords:** *Bougainvillea spectabilis*, antioxidant activity, ham, edible flowers, sensory analysis

## Abstract

In this study, the impact of incorporating *Bougainvillea spectabilis* powder into ham formulation as a potential color replacement for nitrites was evaluated. Three drying methods were proposed to preserve the antioxidant properties of bougainvillea: foam-mat drying, air drying, and oven drying. Antioxidant assays (DPPH, ABTS, and FRAP) assays revealed that the presence of bougainvillea powders enhanced the antioxidant properties and maintained the stability of the ham over 8 weeks of storage at 4 °C. In addition, total polyphenolic content and presence of thiobarbituric acid reactive substances (TBARS) were evaluated and showed higher and lower scores, respectively, in the samples with the incorporation of bougainvillea compared to the control samples, suggesting their potential to replace nitrite salts by providing natural antioxidant protection. Sensorial analysis also revealed no significant differences in sensory attributes in hams with 0.1% bougainvillea powder compared to nitrite samples. The incorporation of the bougainvillea powders in the ham formulation improved the sensorial attributes and consumer overall acceptance even after 8-week cold storage at 4 °C.

## 1. Introduction

Meat and meat products play an important role in human nutrition, acting as a valuable source of bioavailable nutrients, including proteins, iron, minerals, and vitamins [1]. Nitrites (NO_2_^−^) and nitrates (NO_3_^−^) are commonly used as meat formulation additives to improve meat quality. The nitrites not only contribute to the appealing color of meat through their interaction with muscle myoglobin but also improve flavor, exhibit antioxidant properties, and enhance antimicrobial characteristics, thereby extending the shelf life of meat products [2,3,4]. However, the International Agency for Research on Cancer (IARC) has stated that red and processed meat consumption is likely to improve the cancer risk associated with the presence of nitrites, among other issues [5]. These compounds induce the formation of N-nitrosamines and N-nitrosamides, recognized for their impact on carcinogenic and genotoxic processes [3,4]. However, the multiple functions of nitrite and nitrate salts in meat and meat products have prevented their substitution by other food additives, although their presence is generally regulated.

In recent decades, an effort has been made to develop “clean label” products by exploring additive alternatives to reduce the use of nitrites in meat processing. Some vegetables, herbs, spices, fruits, and flowers with antioxidant properties have been studied [4,6]. Pan et al. [7] incorporated bamboo leaf extract into pork ham to prevent nitrite transformation into N-nitrosamines [7]. Ozaki et al. [8] investigated the addition of radish powder and oregano essential oil in fermented cooked pork and beef sausages to enhance the color and inhibit the mesophilic bacteria. However, it did not prevent lipid oxidation effectively. Other examples include the use of celery juice concentrated in ham [9], tomato processing byproducts [10], red wine or red wine and garlic [11], beet root powder [12], pomegranate peel extract [13], and cranberry powder in sausages [14]. However, organoleptic properties need to be further studied.

**Figure 1:**
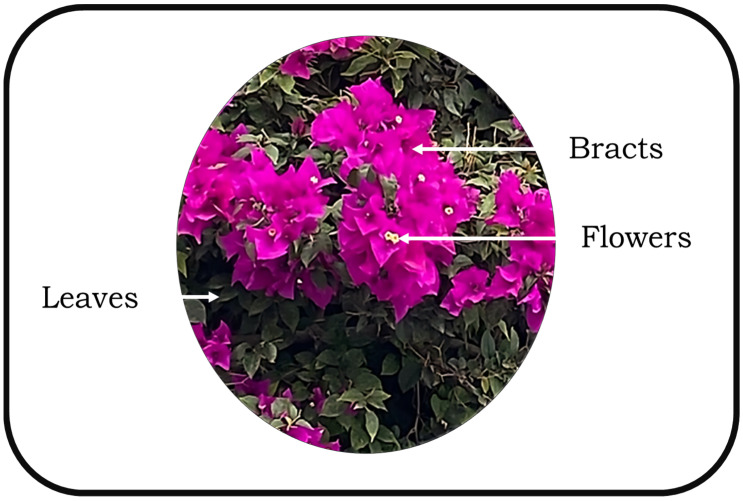
Bougainvillea spectabilis morphology.

*Bougainvillea spectabilis*, commonly known as bougainvillea, is renowned for its colorful bracts (Figure 1) due to the presence of natural coloring pigments such as betalains (betacyanin and betaxanthins), with interesting chemical properties. On the other hand, bougainvillea also contains compounds such as flavonoids, alkaloids, phenols, and tannins, which contribute to its potential as a natural additive [15,16]. These components show strong antioxidant activity and potential anti-inflammatory and anticancer properties and liver-protective effects [17]. Recently, Abdelrahman et al. [18] have reported the antioxidant and antimicrobial activity of phenolic acids, specifically anthocyanins, from bougainvillea and their in vitro inhibitory effect on the viability of certain cancer cells. This flower has been used in Mexican folk medicine as a tea for treating respiratory diseases [16,19,20,21,22,23], suggesting potential benefits for food preservation. Kaushik et al. have described the applications of *Bougainvillea spectabilis* in food products. It has been mentioned that bougainvillea has been incorporated into noodles, pasta, juice, macaroni, frozen desserts, beverages, milk products, tablets, and syrup as the coloring agent. It has been described that this plant could be considered for the food and pharmacy industries [24]. However, it is important to note that comprehensive studies on the safety and efficacy of incorporating bougainvillea extracts into meat products are limited. Further research is essential to understand the specific advantages and potential challenges associated with utilizing *Bougainvillea spectabilis* in the context of meat and meat products.

In this context, an innovative proposal emerges to use *Bougainvillea spectabilis* as an additive in cooked ham, aiming to replace conventional nitrite salts. This study aims to evaluate the impact of different drying methods (air-drying, foam-mat drying, and oven drying) on the preparation of bougainvillea powder and their effect on the physicochemical and sensory properties of the ham, including antioxidant characteristics.

## 2. Materials and Methods

### 2.1. Reagents, Additives, and Solutions

All the reagents employed were of analytical grade and used without further purification. Reagents used in the preparation of the foam mat from bougainvillea, including maltodextrin, hydroxyethyl cellulose, and egg albumin (EA), were obtained from Food Technologies Trading (Mexico City, Mexico). Tween-80 was acquired from J.T. Baker (Phillipsburg, NJ, USA). Additives for ham formulation were coarse marine salt (Altamar, Mexico), polyphosphates (Bekafos Ambsa, Bekarem, Mexico), dextrose (marca), carrageenan (Gelybekam, Bekarem), sodium erythorbate (Bekarem, Iztapalapa, Mexico), sodium nitrite (Sigma-Aldrich, Saint Louis, MO, USA), and dextrose (Food Technologies Trading, Mexico City, Mexico).

For antioxidant activity assays, reagents such as 2,2-Diphenyl-1-picrylhydrazyl (DPPH), 6-hydroxy-2,5,7,8-tetramethylchroman-2-carboxylic acid (Trolox), potassium persulfate, 2,4,6-tris-2-pyridyl-s-triazine (TPTZ), hydrochloric acid, acetic acid, Folin–Ciocalteu reagent, gallic acid, thiobarbituric acid (TBA), 1,1,3,3-tetraethoxypropane (TEP), and methanol (MeOH) were sourced from Sigma Aldrich (St. Louis, MO, USA). ABTS was obtained from Roche Diagnostics (Indianapolis, IN, USA), while trichloroacetic acid (TCA) was acquired from Meyer (Estado de Mexico, Mexico). Iron (II) sulfate, sodium carbonate, and sodium acetate were provided by J.T. Baker (Phillipsburg, NJ, USA), and ferric (III) chloride was purchased from Merck (Darmstadt, Hesse, Germany). For nitrite determination, reagents were as follows: sodium tetraborate, potassium ferrocyanide, zinc acetate, sulfanilamide, sodium nitrite, acetic acid, and naphthyl ethylenediamine (NED). A NED solution was prepared by dissolving 0.2 g of NED in 150 mL of acetic acid solution (15% *v*/*v*). A sulfanilamide solution was prepared by dissolving 0.5 g of the reagent in 150 mL of acetic acid solution (15% *v*/*v*).

All the solutions were prepared with deionized water (Milli-Q Merck, Millipore Darmstadt, Hesse, Germany) with a resistivity of 18.2 MΩ cm or greater.

### 2.2. Preparation of the Bougainvillea Ingredients

Bougainvillea bracts and flowers were carefully collected from *Bougainvillea spectabilis* plants in Pachuca, Hidalgo, Mexico, from January to April 2023. For the preparation of bougainvillea powders, both the flowers and bracts were considered since it has been described that these parts contain the highest concentration of bioactive compounds of interest [15].

In the case of air drying (BA), approximately 100 g of bougainvillea bracts and flowers were weighed and allowed to air dry at room temperature (20–25 °C) in a dark and dry place with good ventilation. These conditions are necessary to prevent and minimize color loss. This drying process typically takes 5 to 7 days. For oven drying (BO), around 100 g of bougainvillea bracts and flowers were weighed and placed in a refractory container. The drying process was carried out in an oven at 65 °C for 4 h.

Finally, the foam-mat drying was applied as the previously described procedure [15]. For this purpose, 25 g of bougainvillea bracts and flowers were combined with 100 mL of distilled water and blended in a food processor. Then, this mixture was whipped for 15 min until frothing using a hand mixer in the presence of 15 g of albumin, 10.0 g of maltodextrin, 2.0 g of hydroxyethyl cellulose, and 2.0 g of Tween-80. The resulting foam was then dried in an oven at 60 °C for 4 h and called BF. After the drying processes, the dried materials BA, BO, and BF were milled in a UDY cyclone sample mill (UDY Corp., Fort Collins, CO, USA) to a particle size of 0.5 mm and preserved in hermetic polyethylene bags in darkness at room temperature until its use.

### 2.3. Cooked Ham Manufacturing

Five ham formulations were designed with the addition of bougainvillea powders plus two control formulations as follows: C, the control with no nitrites nor bougainvillea addition; C-NO_2_, the control with the addition of 150 mg of nitrites kg^−1^ ham; F1, F2, and F3 formulations with 0.05%, 0.1%, and 0.25% of bougainvillea powder in the form of foam BF, respectively; F4 with 0.1% of BA; and F5 with 0.1% of BO. Hams of 2 kg (3 units per formulation) were elaborated at the pilot plant of the Food Chemistry Area in the Universidad Autónoma del Estado de Hidalgo according to regular procedures. Pork leg meat was purchased from a local provider coming from a single farm, manually cleaned from fat and connective tissue, cut into pieces around 5 × 5 cm, mixed, and frozen at −20 °C. The meat was thawed overnight and manually injected with brine prepared to achieve the designed formulation. The formulation of ham consisted of 71.43% pork meat, 2% salt, 0.5% polyphosphates, 0.7% dextrose, 0.75% carrageenan, 0.05% sodium erythorbate, and 24.57% water and the bougainvillea additive or nitrites according to the formulation. Brine was prepared using the following formulation (86% cold water, 7% salt, 1.75% polyphosphates, 2.45% dextrose, 2.62% carrageenan, and 0.175% sodium erythorbate) and injected at 40%. Bougainvillea powders were added to the brine before injection to reach the designed concentrations in the final product. After injection, the hams were tumbled and massaged for 10 min every hour at refrigeration temperature for 14 h. After that, the meat was placed in polyethylene bags inside cylindrical plastic molds. The hams were cooked until a core temperature of 67 °C in a Rational 20-2/1 oven unit (Landsberg am Lech, Germany), cooled in ice water for 2 h, and refrigerated at 4 °C. After 24 h, hams were unmolded and sampled for physicochemical and sensorial analysis, while the remaining product was vacuum packed and stored refrigerated at 4 °C for 8 weeks to follow color and antioxidant evolution at 4 and 8 weeks.

### 2.4. Physicochemical Properties

The cooking yield of the process was gravimetrically calculated as the percentage of ham weight after unmolding related to the weight before cooking. The pH of each sample of each formulation was measured using a digital pH meter (HI 99161, Hanna Instruments, Ronchi Di Villafranca Padovana, Italy) equipped with a glass probe for penetration. Water activity was determined in an AquaLab 3TE (Decagon, WA, USA). To determine drip loss, a 100 g sample was placed in a plastic bag vacuum-sealed and kept at 4 °C. After 8 weeks, the sample was dried with absorbent paper and weighed. The amount of drip was expressed as the percentage of drained water related to the initial weight. The water holding capacity (WHC) was measured based on the filter paper press method described by Steen et al. [25]. A weight of 0.3 g of ham was placed on a Whatman No. 2 filter paper between two plexiglass plates, and a weight of 1 Kg was placed over it for 5 min. The expelled fluid was absorbed by the filter paper, forming an outer circle around the inner circle formed by the meat. Both areas were measured with the help of the software ImageJ ver. 1.54f (NIH, 2023) [26], and the WHC was expressed as the ratio area of water expelled/area of meat (cm^2^/cm^2^) [27].

Moisture content in hams was determined using the moisture determination method outlined by the Association of Analytical Communities (AOAC, 2003) [28] in their standard 23.003:2003. This involved weighing samples of 2.000 ± 0.001 g each, which were subsequently dried in an oven at 105 °C until a constant weight was reached.

Crude fat was extracted by the Soxhlet procedure with petroleum ether at 80 °C and determined gravimetrically according to the AOAC 960.39 standard [28]. Protein content was also determined by the International Organization for Standardization standard ISO Kjeldahl method 937:1978 (ISO, 1978) [29]. Nitrogen was determined after digestion of a 1 g sample with sulfuric acid, distilled with NaOH, recovering the liberated ammonia in boric acid in a Gerhardt distillation unit (Königswinter, Germany), and titrated with HCl 0.1 N. Protein was calculated from total nitrogen concern using the conversion factor of 6.25. These above physicochemical determinations were obtained in triplicate.

Nitrite content in the ham formulations was determined using the Griess test [30,31]. Before analysis, the ham samples were homogenized and deproteinized following the method described by Belluci et al. [32]. Briefly, a 5.0 g sample was mixed with 2.5 mL of 5% sodium tetraborate and 25 mL of hot water (85 °C), then heated for 15 min. After transferring to a 100 mL flask, an additional 25 mL of hot water was added, and the mixture was cooled. Moreover, 2.5 mL of 15% potassium ferrocyanide and 2.5 mL of a 30% zinc acetate solution were incorporated into the mixture, and the volume was adjusted to 100 mL. Afterwards, sulfanilamide and NED were added to 5.0 mL of the filtrate, and the absorbance was measured at 540 [31,32]. Measurements were performed in triplicate. Spectrophotometric measurements were conducted with a UV/Vis spectrometer, the Perkin Elmer Lambda 40 (Waltham, MA, USA), employing the Perkin Elmer UVWinLab software. Results were expressed as mg NO_2_ kg^−1^.

CieLab color parameters L*, a*, and b* (where L* represents color lightness, a* denotes redness, and b* indicates yellowness) were measured at four randomly selected points on the sample surface after cutting on the middle of the sample with a portable colorimeter Hunter Lab miniScan EZ 4500L (HunterLab, Reston, VA, USA) under D65 illuminant and 10° observer angle. Tiles in black and white were used to calibrate the equipment. The mean of the four measurements was used per sample. The three hams per formulation were measured, and the means per ham were used for statistical analysis. The color was measured at 0, 4, and 8 weeks.

### 2.5. Sensory Evaluation

A 7-point hedonic test was employed for the sensory evaluation of ham formulations. Samples were sliced 2 h before the tasting (2 mm thick), wrapped in aluminum foil, and kept at room temperature. Thirty minutes prior to the sensorial session, slices were cut into rectangular pieces of 6 × 3 cm. Seventeen untrained but accustomed to participating in meat products sensory analysis panelists conducted the test in one session with the seven samples labeled by three-digit numbers and presented them randomly. The hedonic scores ranged from 1 to 7, as follows: very unpleasant (1), quite unpleasant (2), slightly unpleasant (3), acceptable (4), slightly pleasant (5), quite good (6), and excellent (7). The test included color, odor, taste, and overall acceptability. Hams were evaluated 48 h after the cooking process. Plastic dishes were used to present the samples to the panelists, and water and bread were provided to cleanse the palate from residual flavors between tastings [33].

### 2.6. Antioxidant Activity

Methanolic extracts from the samples were prepared to evaluate the antioxidant activity. Two hundred grams of ham sample were ground with a food processor, and 2.0 g were placed in a polypropylene tube with 5.0 mL of methanol. The mixture was vortexed for 10 min to obtain the methanolic extract [15]. The mixtures underwent ultrasound extraction for 10 min, followed by centrifugation at 2200 rpm and filtration using Whatman paper (Whatman 41). Each sample was obtained in triplicate.

The antioxidant activity of the ham formulations was evaluated considering DPPH and ABTS scavenging activity, as well as ferric-reducing antioxidant activity (FRAP). Additionally, the assessment included the determination of total polyphenolic content (TPC) and the measurement of lipid oxidation. This approach facilitated a comprehensive evaluation of the oxidative stability of the ham formulations throughout distinct storage intervals (weeks 0, 4, and 8). Spectrophotometric measurements were conducted with the UV/Vis spectrometer Perkin Elmer Lambda 40 (Waltham, MA, USA) employing the Perkin Elmer UVWinLab software(version 6.2).

The DPPH method was performed as described by Rivero-Perez et al. [34]. This procedure involved monitoring the decrease in absorbance at 515 nm when the DPPH radical is exposed to the presence of antioxidant species in the ham formulations. For DPPH radical scavenging activity, methanolic extracts of the samples were mixed with a DPPH solution, and the subsequent decrease in absorbance was quantified. ABTS radical scavenging activity was determined by measuring the reduction in the number of ABTS radical cations (ABTS^+●^) in the presence of the sample extracts; the change in absorbance was measured at 734 nm. The results of DPPH and ABTS methodologies were expressed as inhibition percentages. All extracts were prepared using the same sample amount, and the antioxidant activity evaluation was performed with a consistent extract volume of 20 µL and a radical solution volume of 980 µL in each case [33].

The FRAP method was assessed by quantifying the reduction of a ferric complex to its ferrous form according to Benzie et al. [35]. The concentration of antioxidant compounds is related to the increase in the absorbance at 593 nm. The results were expressed as mmol FeSO_4_●100 g^−1^.

In addition to the evaluation of antioxidant activity, the total polyphenolic content (TPC) was determined using the Folin–Ciocalteu method. In this process, a 2 g sample reacted with Folin–Ciocalteu reagent, and the resultant blue color was measured spectrophotometrically (750 nm), with gallic acid as the standard [34]. The results were expressed as mg_gallic acid_ g^−1^.

Lipid oxidation was evaluated by the quantification of thiobarbituric acid reactive substances (TBARS). Following the methodology outlined by Vyncke with slight modifications [36]. Briefly, two grams of a ground sample are mixed with 10 mL of 5% TCA in a Falcon tube, then homogenized on ice for 2 min using an Ultraturrax T-18 (IKA, Wilmington, NC, USA). The samples are centrifuged at 3500 rpm for 10 min after freezing for 10 min to precipitate proteins. The supernatant is filtered, and 5 mL of extract is transferred to a Falcon tube, with dilutions made if necessary. After adding 5 mL of TBA, the sample is vortexed, incubated at 97 °C for 40 min, cooled, and placed in an ultrasonic bath for 15 min. The absorbance is measured at 532 nm. TBARS values were expressed as mg MDA kg^−1^, and the progression of TBARS, indicative of lipid oxidation, was monitored during storage.

### 2.7. Statistical Analyses

The data from color and antioxidant activity were evaluated with a two-factor analysis of variance (ANOVA) (treatment and storage time), while data from the other physicochemical parameters were evaluated by a one-way ANOVA. Tukey’s test was used to compare the mean values when the ANOVA was significant (*p* < 0.05). Regarding the sensory analysis, panelists were considered as a random effect (each panelist tasted samples from all formulations in a single session). Statistical analyses were performed using the Statgraphics Centurion XVI version 16.1.03 (32-bits) (StatPoint Technologies, Inc., Warrenton, VA, USA).

## 3. Results and Discussion

### 3.1. Physicochemical Results

The cooking yield ranged between 94.3 and 97.3% without significant differences (*p* > 0.05) between formulations. No significant differences (*p* > 0.05) were found in aw (0.976–0.983) and small differences were detected in pH (Table 1), while WHC and moisture were significantly affected (*p* < 0.05) by the inclusion of bougainvillea ingredient, especially in the form of foam. Water constitutes approximately 75% of the total weight in meat products, and its retention capacity, known as water holding capacity (WHC), is a crucial parameter for ensuring meat quality [37]. The sample with the higher proportion of bougainvillea accompanied by foam material (F3) presented the lowest significant moisture content (74.74 ± 1.86%) and the highest water holding capacity since these samples showed less water expelled after compression (0.59 ± 0.05). F1 and F2 samples, also with the colorant as foam but with less concentration of bougainvillea, also presented high water holding capacity despite the moisture being similar to the controls and samples with BA and BO colorant. The albumin added in the process of foaming also contributed to a significant increase (*p* < 0.05) in the protein percentage in formulations F2 and F3. Fat content ranged from 1.44 to 2.80%, with small differences between samples attributed to the heterogeneity of raw material.

Regarding drip loss evaluation, protein oxidation has been implicated in reducing water-holding capacity in meat products [37]. After 8 weeks of cold storage, drip loss was measured, yielding values lower than 4% in all cases. F2 and F3 formulations presented the lowest values (2.59 and 2.91%, respectively), likely attributable to the addition of egg albumin during the foam-mat drying process used to produce the bougainvillea powder (BF).

After conducting the evaluation of nitrite content using the modified Griess method [32], it was determined that there was no presence of nitrites in the formulations containing the bougainvillea powders nor the control; just C-NO_2_ presents a residual concentration of 41.60 ± 1.93 mg_NO2_ kg^−1^. This finding confirms that the bougainvillea does not contribute to the nitrite levels in the ham formulations. This supports the potential use of bougainvillea as a safe and natural ingredient in food products aimed at reducing reliance on synthetic additives.

### 3.2. Instrumental Color and Sensory Results

**Figure 2:**
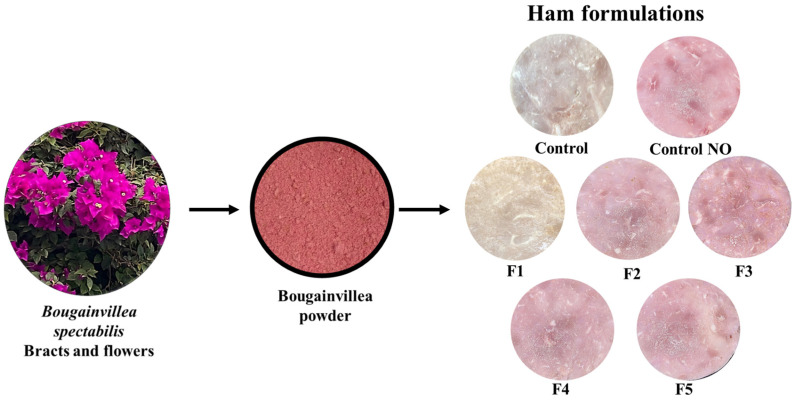
*Bougainvillea spectabilis* powder and ham formulations.

The use of bougainvillea powders in the ham formulations provides a reddish color while offering antioxidant properties and replacing the presence of nitrites (see Figure 2).

According to the color results, formulations F4 and F5 presented L values with no significant difference from the control sample with nitrites, although a* values were significantly lower while b* values were significantly higher than the recorded for C-NO_2_. In fact, F4 and F5 presented the most similar color to the nitrite control sample (62.49 ± 0.53). The F3 formulation with the highest amount of bougainvillea colorant exhibited a similar a* value (8.30 ± 0.56) to the nitrite sample but with significantly higher values of L* and b*. On the contrary, F1 and F2 samples presented a paler color similar to control samples without nitrites (Table 2).

During cold storage, there were consistent slight variations in color, especially in F1-F5 formulations. While a* and L values hardly changed in C-NO_2_ and b* scores slightly increased, indicating that color remained stable during preservation, in the case of F1–F5 samples a significant increase in a* was noticed (*p* > 0.05). Also, a significant decrease in b* values was observed in F1–F5 samples, improving the reddish color of samples, making them more like C-NO_2_, and proving that bougainvillea bracts and flowers can provide a reddish color to cooked ham, replacing nitrites. Similar efforts have been described to incorporate natural colorants with antioxidant activity, such as betacyanins and betaxanthins, in ham formulations. The incorporation of red radish, red beetroot, and hibiscus in cooked ham formulations was proposed by Dias et al. [38]. However, only red beetroot provided a color closer to the intended, but color stability was not evaluated. Natural pigments such as betalains tend to fade out with time because of oxidation reactions, but in this case, it seems that the antioxidant properties of the bougainvillea could be contributing to stabilizing the color [39].

These physical color properties were confirmed by the sensory panel results. According to Table 3, the formulations best evaluated in the visual aspect were samples with nitrites and the formulations with the bougainvillea colorant dried by air (F4) and oven (F5). This color perception was also transferred to the overall acceptance, which was higher in the same samples with no significant differences between them. The odor and the taste were not significantly affected (*p* > 0.05) by the inclusion of the bougainvillea. Odor and taste did not represent significant differences among the different formulations.

### 3.3. Antioxidant Results

Although the antioxidant properties of bougainvillea have been previously studied [15,40,41,42], its incorporation as an additive in meat products has not been described. After the preparation of the formulations, analyses of antioxidant activity were conducted using DPPH, ABTS, and FRAP methods, along with the assessment of total polyphenolic content (TPC) and lipid oxidation (TBARS). This approach allowed the evaluation of the oxidative stability of the ham formulations over various storage periods (weeks 0, 4, and 8) at 4 °C.

**Figure 3:**
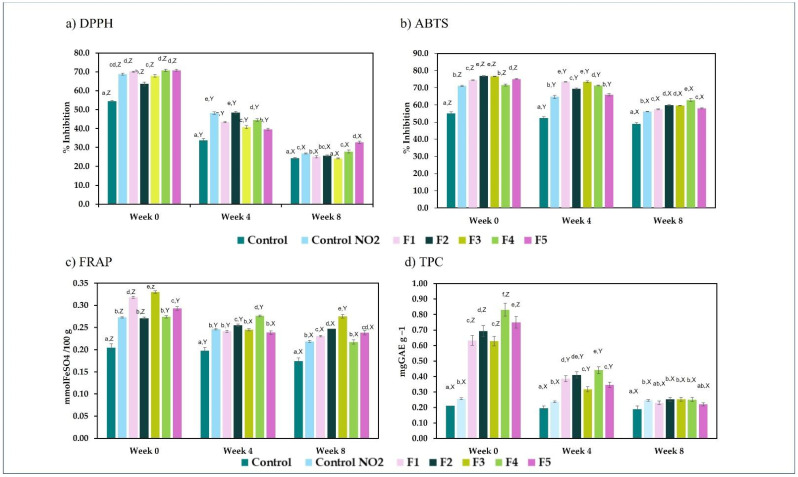
Effect of the addition of bougainvillea powder on the antioxidant activity measured in ham with (**a**) DPPH method, (**b**) ABTS method, (**c**) FRAP method, and (**d**) total polyphenolic content method. a–f mean values for each week with different letters differ significantly (*p* < 0.05), X–Z mean values for each formulation with different letters differ significantly (*p* < 0.05).

Control samples showed the presence of antioxidant compounds due to the addition of sodium erythorbate. Antioxidative activity methods showed that ham samples with bougainvillea powders exhibited higher inhibition percentage values compared to the control sample (Figure 3). The incorporation of bougainvillea powder in ham formulations either matches or enhances the antioxidant capacity compared to the formulation containing nitrite salts. In the DPPH assay, the formulations containing the bougainvillea powders presented a significant difference (*p* < 0.05) compared with the control. The samples exhibited an inhibition percentage of 63.6–70.8%, while the control samples were 54.0%. Similar values were observed in ABTS (71.5–76.6%) during week 0.

Nevertheless, the antioxidant effect significantly diminished during cold storage (*p* < 0.05) in all formulations, independently of adding nitrites or the natural colorant. After 8 weeks of storage, DPPH values decreased to values between 24.12% and 32.98% (24.26% for the control sample and 26.48% for the nitrite sample). Samples F5 showed significant differences (*p* < 0.05) with the control and nitrite control showing an inhibition percentage of 32.64%. For the ABTS assay, a smaller decrease in inhibition percentages was observed, with values ranging from 48.98% to 62.89%. In this case, formulation F4 presented the highest ABTS value (62.89%), while the control and nitrite samples showed the lowest values (48.98% and 56.02%, respectively). All the formulations, including bougainvillea, presented significant differences with the control. DPPH and ABTS results indicate greater stability of polar compounds.

Related to FRAP results, formulations F1 to F5 exhibited more stable antioxidant activity over the 8-week storage period, with smaller decreases in activity than the control, suggesting that bougainvillea contributes to maintaining antioxidant stability. While storage typically leads to the degradation of natural reducing compounds, the control samples showed less antioxidant protection over time. These findings highlight the effectiveness of bougainvillea powders as a natural antioxidant alternative, offering extended oxidative protection compared to synthetic additives like sodium erythorbate (C) and nitrite salts (C-NO_2_).

The antioxidant activity of plants is related to their bioactive content [15]. Orozco-Villafuerte et al. [43] observed a direct correlation between phenolic compounds and antioxidant activity of *Bougainvillea spectabilis*. In this case, the content of phenolic compounds (TPC) significantly increased (*p* < 0.05) in the presence of bougainvillea additives in ham. The higher content of TPC was found in the F4 formulation (0.831 ± 0.024 mg_GAE_ kg^−1^). The lowest concentration was found in the control sample (0.211 ± 0.006 mg_GAE_ kg^−1^). In this case, the presence of polyphenolic compounds in the control and nitrite control was observed. This information agrees with the information described by Bešlo et al., who described a growing interest in the use of by-products in animal nutrition with high concentrations of polyphenols. They described this diet as contributing to greater stability of meat to fatty acid oxidation of meat products for human consumption [44,45].

**Figure 4:**
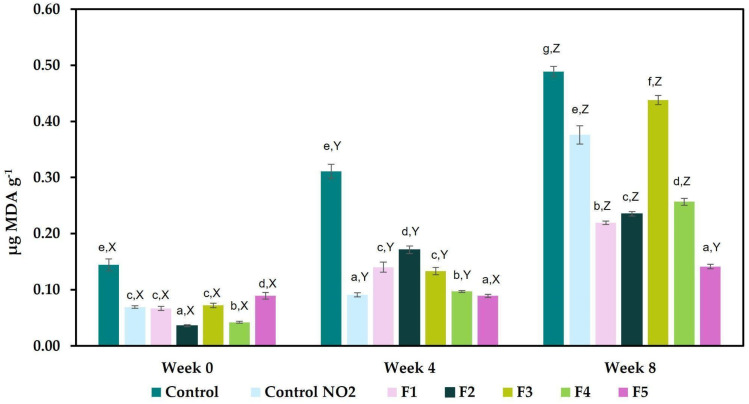
Effect of the addition of bougainvillea on the content of thiobarbituric acid reactive substances (TBARS assay) in mg MDA/kg. a–g mean values for each week with different letters differ significantly (*p* < 0.05). X–Z mean values for each formulation with different letters differ significantly (*p* < 0.05).

The presence of bioactive compounds with antioxidant activity influences lipid stability, which is related to meat quality since it prevents protein oxidation, discoloration, and rancidity [46]. This parameter was assessed with the thiobarbituric acid reactive substances method (TBARS). The addition of bougainvillea powders decreased lipid oxidation (F2 presented the significant lowest value of 0.0360 μg_MDA_ g^−1^) compared with the control (0.1440 μg_MDA_ g^−1^) and nitrite control samples (0.0691 μg_MDA_ g^−1^). Generally, the TBARS values in ham increased with cool storage; however, because of the presence of bougainvillea, this effect was observed to a lesser extent (Figure 4). In this study, all the formulations have acceptable levels during the evaluation period (<0.500 μg_MDA_ g^−1^).

The analysis of TBARS shows that the addition of bougainvillea in the formulations significantly reduces oxidative processes in meat. The TBARS test, which measures lipid peroxidation and thus oxidative rancidity, indicated that formulations with bougainvillea had lower values compared to the control, which exhibited the highest TBARS concentration; at week 0, all the formulations had values <0.150 µg_MDA_ g^−1^. This suggests that bougainvillea is effective in mitigating oxidative damage, competing with the antioxidative effect provided by nitrites. Several plants, such as celery or Swiss chard powder and beetroot or barberry extract, have been considered alternative sources of nitrites, but some of them contain nitrate, which can be transformed into nitrites [3]. In this case, the absence of nitrite presence in the cooked ham with bougainvillea, the improvement of color, and antioxidant properties make *Bougainvillea spectabilis* a good candidate to be used in cooked meat products, but always as one more strategy within a hurdle technology that ensures a microbiologically safe product.

## 4. Conclusions

This study demonstrates that incorporating bougainvillea powder into ham formulations provides a viable natural color alternative to nitrites, maintaining cooking yield and physicochemical properties such as water holding capacity and moisture content. The bougainvillea powder, regardless of the drying process to obtain it, improved the antioxidant stability of the ham, as evidenced by increased total polyphenolic content and superior performance in DPPH, ABTS, and FRAP assays. Sensory evaluation confirmed that the bougainvillea-treated hams retained desirable color, odor, and taste, with formulations F4 and F5 achieving high visual and overall acceptance scores. Importantly, no nitrites were detected in any formulation, affirming the potential of *Bougainvillea spectabilis* as a natural color ingredient for healthier and more sustainable meat products within a set of measures that ensure the safety of the product.

## Figures and Tables

**Table 1:** Physicochemical data obtained from ham formulations (N = 3).

Formulation	pH	WHC	Drip Loss (%)	Moisture Content (%)	Fat (%)	Protein (%)
C	5.60 ± 0.01 ^a^	0.69 ± 0.01 ^b^	2.83 ± 0.61	76.45 ± 1.12 ^ab^	1.81 ± 0.34 ^a^	14.73 ± 0.40 ^a^
CNO_2_	5.69 ± 0.01 ^d^	0.69 ± 0.03 ^b^	3.57 ± 0.91	76.33 ± 0.66 ^ab^	2.80 ± 0.17 ^c^	14.51 ± 0.76 ^a^
F1	5.67 ± 0.02 ^c^	0.62 ± 0.02 ^a^	3.56 ± 0.29	77.39 ± 0.49 ^b^	1.59 ± 0.08 ^a^	14.74 ± 0.35 ^a^
F2	5.67 ± 0.02 ^c^	0.62 ± 0.01 ^a^	2.59 ± 0.37	77.35 ± 0.13 ^b^	1.89 ± 0.26 ^ab^	15.92 ± 0.57 ^b^
F3	5.59 ± 0.01 ^a^	0.59 ± 0.05 ^a^	2.91 ± 0.26	74.74 ± 1.86 ^a^	2.36 ± 0.39 ^bc^	17.07 ± 0.54 ^c^
F4	5.66 ± 0.02 ^c^	0.71 ± 0.02 ^b^	3.16 ± 0.61	77.31 ± 1.38 ^b^	1.66 ± 0.31 ^a^	14.69 ± 0.70 ^a^
F5	5.63 ± 0.02 ^b^	0.72 ± 0.03 ^b^	3.11 ± 0.38	77.75 ± 0.64 ^b^	1.44 ± 0.39 ^a^	14.28 ± 0.69 ^a^

^a–d^ means in the same column with different superscripts are significantly different (*p* < 0.05).

**Table 2:** Color parameter results (L, a*, and b*) during storage period (0, 4, and 8 weeks).

		Storage (Weeks)		
	Formulation	0	4	8
L	C	64.06 ± 0.51 ^cd,X^	63.49 ± 0.26 ^d,X^	66.62 ± 0.36 ^d,Y^
	C-NO_2_	62.49 ± 0.53 ^a^	61.90 ± 0.56 ^c^	62.55 ± 0.40 ^b^
	F1	64.32 ± 0.23 ^cd,Z^	62.70 ± 0.32 ^cd,Y^	63.77 ± 0.25 ^c,X^
	F2	64.40 ± 0.57 ^d,Y^	62.38 ± 0.31 ^c,X^	63.93 ± 0.08 ^c,Y^
	F3	63.57 ± 0.34 ^bc,Z^	59.22 ± 0.75 ^a,X^	61.20 ± 0.40 ^a,Y^
	F4	62.83 ± 0.40 ^ab,Y^	61.07 ± 0.59 ^b,X^	63.49 ± 0.47 ^c,Y^
	F5	62.43 ± 0.47 ^a,Z^	59.66 ± 0.23 ^a,X^	61.00 ± 0.09 ^a,Y^
a*	C	4.62 ± 0.42 ^a,X^	5.90 ± 0.21 ^a,Y^	4.97 ± 0.08 ^a,X^
	C-NO_2_	8.35 ± 0.39 ^d^	8.60 ± 0.83 ^c^	9.27 ± 0.93 ^d^
	F1	5.24 ± 0.20 ^a,X^	6.10 ± 0.32 ^a,Y^	6.08 ± 0.04 ^a,Y^
	F2	6.13 ± 0.15 ^b,X^	7.12 ± 0.31 ^b,Y^	8.64 ± 0.02 ^cd,Z^
	F3	8.30 ± 0.56 ^d,X^	10.63 ± 0.26 ^d,Y^	10.32 ± 0.23 ^e,Y^
	F4	6.54 ± 0.41 ^b,X^	8.43 ± 0.28 ^c,Y^	8.37 ± 0.24 ^c,Y^
	F5	7.50 ± 0.29 ^c,X^	8.61 ± 0.80 ^c,Y^	11.44 ± 0.06 ^f,Z^
b*	C	15.93 ± 0.56 ^e^	15.56 ± 0.65 ^d^	16.05 ± 0.40 ^e^
	C-NO_2_	11.19 ± 0.28 ^a,X^	13.09 ± 0.38 ^b,Y^	13.02 ± 0.81 ^bc,Y^
	F1	14.97 ± 0.31 ^cd,X^	16.01 ± 0.25 ^d,Y^	14.89 ± 0.35 ^d,X^
	F2	15.18 ± 0.59 ^d,Y^	14.52 ± 0.31 ^c,Y^	13.26 ± 0.42 ^c,X^
	F3	14.41 ± 0.14 ^c,Y^	14.17 ± 0.08 ^c,Y^	12.32 ± 0.21 ^b,X^
	F4	13.44 ± 0.38 ^b,Y^	13.41 ± 0.53 ^b,Y^	11.16 ± 049 ^a,X^
	F5	12.92 ± 0.11 ^b,Y^	12.36 ± 0.30 ^a,Y^	10.74 ± 0.63 ^a,X^

Results are expressed as mean value ± standard deviation (N = 3). ^a–f^ means in the same column with a different letter are significantly different (*p* < 0.05). ^X–Z^ means in the same row with a different letter are significantly different (*p* < 0.05).

**Table 3:** Sensory panel results.

Formulation	Visual Aspect	Odor	Taste	Overall Acceptance
C	3.35 ± 1.37 ^a^	4.53 ± 1.07 ^a^	4.94 ± 1.14 ^a^	4.88 ± 1.05 ^ab^
C-NO_2_	6.24 ± 1.15 ^d^	4.53 ± 1.62 ^a^	5.41 ± 1.42 ^a^	5.47 ± 1.42 ^b^
F1	4.29 ± 1.40 ^b^	4.82 ± 1.19 ^a^	5.12 ± 1.05 ^a^	4.88 ± 0.83 ^ab^
F2	4.65 ± 1.32 ^bc^	4.29 ± 1.10 ^a^	5.06 ± 1.25 ^a^	4.88 ± 0.93 ^ab^
F3	4.59 ± 1.33 ^bc^	4.24 ± 1.56 ^a^	4.59 ± 1.37 ^a^	4.29 ± 1.65 ^a^
F4	5.65 ± 1.41 ^d^	4.65 ± 1.32 ^a^	5.24 ± 1.09 ^a^	5.24 ± 1.09 ^b^
F5	5.41 ± 1.28 ^cd^	4.76 ± 1.25 ^a^	5.18 ± 1.63 ^a^	5.18 ± 1.33 ^b^

Results are expressed as mean value ± standard deviation. ^a–d^ means in the same column with a different letter are significantly different (*p* < 0.05).

## Data Availability

The original contributions presented in the study are included in the article, further inquiries can be directed to the corresponding author.
